# Characterisation of Fluid Administration in Burn Shock—A Retrospective Cohort Analysis

**DOI:** 10.3390/ebj6020035

**Published:** 2025-06-10

**Authors:** Marianne Kruse, Ida Katinka Lenz, David Josuttis, Philip Plettig, Klaus Hahnenkamp, Denis Gümbel, Claas Güthoff, Bernd Hartmann, Martin Aman, Marc Dominik Schmittner, Volker Gebhardt

**Affiliations:** 1 Department of Anesthesiology, Intensive Care and Pain Medicine, BG-Klinikum Unfallkrankenhaus Berlin, 12683 Berlin, Germanymarc.schmittner@ukb.de (M.D.S.); volker.gebhardt@ukb.de (V.G.); 2 Department of Anesthesiology, University Medicine Greifswald, 17475 Greifswald, Germany; klaus.hahnenkamp@med.uni-greifswald.de; 3 Department of Orthopedics, Trauma Surgery and Rehabilitative Medicine, University Medicine Greifswald, 17471 Greifswald, Germany; denis.guembel@ukb.de; 4 Department of Trauma Surgery and Orthopedics, BG Hospital Unfallkrankenhaus Berlin gGmbH, Warener Straße 7, 12683 Berlin, Germany; 5 Center for Clinical Research, BG Klinikum Unfallkrankenhaus Berlin gGmbH, 12683 Berlin, Germany; claas.guethoff@ukb.de; 6 Burn Center, Department of Plastic Surgery, BG Klinikum Unfallkrankenhaus Berlin, 12683 Berlin, Germany; bernd.hartmann@ukb.de; 7 Clinic for Hand, Replantation, and Microsurgery BG Klinikum Unfallkrankenhaus Berlin and Chair of Hand, Replantation, and Microsurgery at the Charité University Medicine Berlin, 10117 Berlin, Germany; martin.aman@ukb.de; 8 Medical Faculty Mannheim of Heidelberg University, Ruprecht-Karls-University Heidelberg, 68167 Mannheim, Germany

**Keywords:** severe burn injury, fluid resuscitation, fluid creep, burn shock

## Abstract

Background: Finding the optimal amount of fluid is a major challenge in burn shock. Although there is evidence that a restrictive fluid regime is beneficial, current practice shows fluid resuscitation still well above recommendations. The extent of trauma, pre-hospital care and the patient’s pre-existing conditions influence requirements. Methods: We analysed outcomes and influencing factors of fluid regimes in a retrospective cohort study including 90 severely burnt patients resuscitated with the same protocol. Results: The mean amount of fluids in the first 24 h was 6.5 mL/kg bodyweight (BW)/% total burn surface area (TBSA). A total of 14% received restrictive (<4), 34% received liberal (4–6) and 51% received excessive (>6) mL/kgBW/%TBSA fluids. There was no difference regarding mortality, age, complications, organ failure, inhalation injury or full-thickness burns in the groups. Patients with excessive fluid therapy had a significantly lower ABSI score (9 vs. 11, *p* = 0.05) and TBSA (35 vs. 51%, *p* < 0.001), while patients with a restrictive fluid therapy needed fewer incidences of surgery to cover burn wounds (3.5 vs. 9.0 vs. 7.0, *p* = 0.008). History of liver disease or alcohol abuse tended to indicate excessive fluid administration. Patients with pre-existing heart failure received restrictive fluid therapy (23 vs. 3 vs. 4%, *p* = 0.03). Conclusions: Individualised, timely therapy monitoring is as essential as identifying patients with a higher or lower fluid requirement. Excessive fluid resuscitation had fewer deleterious consequences in complications than expected but seems to influence wound healing. Awareness of circumstances that prompt deviations from recommended fluid rates remains elementary.

## 1. Introduction

The assessment of the optimal amount of fluid to be administered to patients after severe burn injury is still one of the main challenges during burn shock and has been the subject of several scientific investigations [1,2,3]. The pathophysiology of burn shock is a combination of fluid and protein loss to extravascular tissues as well as cardiac depression due to inflammatory response. There are damage-associated patterns as well as pathogen-associated patterns at the beginning of pathophysiological cycles. This process leads to an excessive release of mediators like cytokines or NO, followed by immune dysfunction, hypermetabolism and hyperinflammation [4,5]. The result is a cascade of increased vascular permeability, disruption of collagen, increased hydrostatic pressure and systemic edema, not limited only to burnt tissue [6].

Combined with burn-induced cardiac depression and the necessity of maintaining circulation to avoid multi-organ dysfunction syndrome (MODS), the result is an immense need for fluids, which is difficult to estimate and leads to an individual demand of fluid resuscitation in every patient.

Apart from formula-based fluid calculation and symptom-targeted therapy like haemodynamic parameters or values of organ function [7,8], there is still a lack of clarity about the underlying individual causes that can lead to a general need for more or less fluid during the burn shock phase. There are several causes named, for example, the existence of inhalation injury or high-voltage burns [9,10,11] as well as a high percentage of affected tissue [12], which increase the fluid requirement. Even though there has been a lot of effort made to clarify this topic, it remains unclear what the ultimate consequences of therapy that misses the target are. There are indications that either too little or too much fluid in the first 24 h after trauma can cause organ dysfunction [13,14,15]. The literature indicates that fluid overtreatment, the so-called “fluid creep”, is very likely to cause more damage than strictly restrictive fluid administration [16,17,18,19]. For this reason, alternative formulas to the gold-standard Parkland–Baxter formula have been developed, which, like the modified Brooke formula, only target half the fluid which is determined by using the Parkland–Baxter formula during burn shock in the first 24 h. However, in clinical practice, there is often more fluid administered than initially calculated and recommended [20]. Future research should focus on optimising fluid therapy and understanding underlying factors [21].

In this retrospective evaluation of a large cohort of severely burnt patients, we tried to identify causes for the different fluid requirements. We investigated whether fluid management is associated with patient outcomes and analysed patient- and trauma-related factors to improve the predictive estimation of fluid requirements of burn patients and to avoid future under- and over-therapy.

## 2. Methods

We conducted a retrospective analysis of patients admitted to our burn centre between 2014 and 2019. Patients with flame injuries, electrical burns and scald-type burns were included. The study was approved by the local Ethics Committee (Ethik-Kommission Ärztekammer Berlin) on 27 January 2021 (Eth-44/20) and registered at the German Clinical Trial Registry (ID: DRKS00033516). According to federal law, there was no requirement of written patient consent.

**Figure 1:**
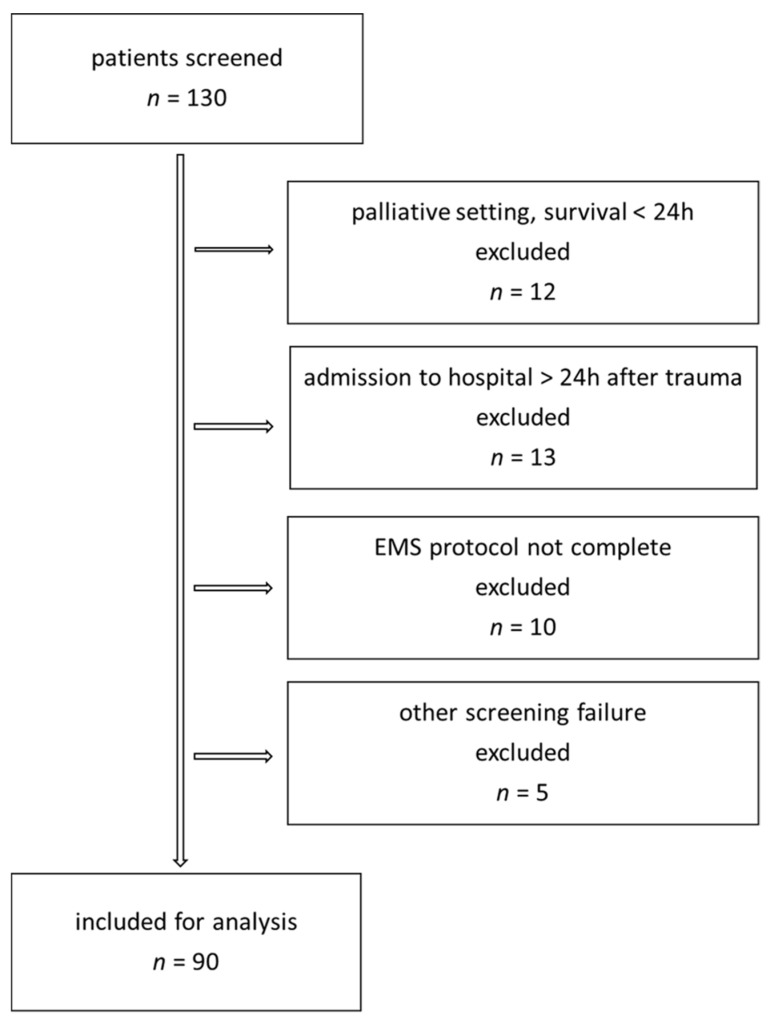
Patient inclusion chart.

We included adult patients with a >20% burned surface area. Exclusion criteria were toxic epidermic necrolysis, primary palliative care treatment or admission to hospital more than 24 h after trauma (Figure 1). All resuscitations were carried out according to our hospital standard using Ringer’s acetate solution. Fluid requirement was calculated according to the Parkland–Baxter formula. We used measured body weight and estimated 4 mL/kgBW/%TBSA with planned application of half of the fluids in the first 8 h. Fluid rate was adjusted based on haemodynamic parameters (MAP > 65 mmHg) and diuresis (≥0.5 mL/kg/h) and central venous oxygen saturation (ScVo_2_ > 70%) at the physician’s discretion. Adjustments for patients with inhalation or high-voltage injury were only made based on individual requirements. Implementation was carried out by both physicians and nurses. Human albumin administration started 24 h after trauma. The first choice of vasopressor was noradrenaline. The protocol included a four-hourly measurement of intra-abdominal pressure in all patients with >20%TBSA.

Clinical data of patient status at time of admission was extracted retrospectively from paper-based patient records and from the digital patient ICU chart (ICM, Dräger, Luebeck, Germany). Also, we used the available data from the hospital data management system (medico, Cerner Health Services, Berlin, Germany). The Baux score was calculated as previously published [22]. The Abbreviated Burn Severity Index (ABSI) and clinical parameters and scores used for definition of organ failure were also extracted from the medical records (Table 1).

Data was extracted in a pseudonymous manner and imported into SPSS (27, IBM, Armonk, NY, USA).

We conducted an exploratory data analysis mainly focusing on cohort description. If not indicated differently, metric study parameters are presented as median with interquartile range (IQR). For comparisons between two groups, the nonparametric Mann–Whitney U test was performed. For comparisons between three or more groups, the Kruskal–Wallis test was used. To compare the calculated fluid volume according to the Parkland–Baxter formula to the administered fluid, we used a paired *t*-test. Categorical variables are shown as counts and percentages. Frequencies were compared with the chi–square test or Fisher’s exact test when expected cell frequencies were <5. In order to visualise metric patient and burn wound characteristics, receiver operating curves were used. To adjust for confounders, we performed logistic regression analyses. A two-sided *p*-value of <0.05 was considered statistically significant.

For group comparisons of numbers of required surgeries, we excluded all patients who died before their surgical treatment could be completed.

## 3. Results

**Figure 2:**
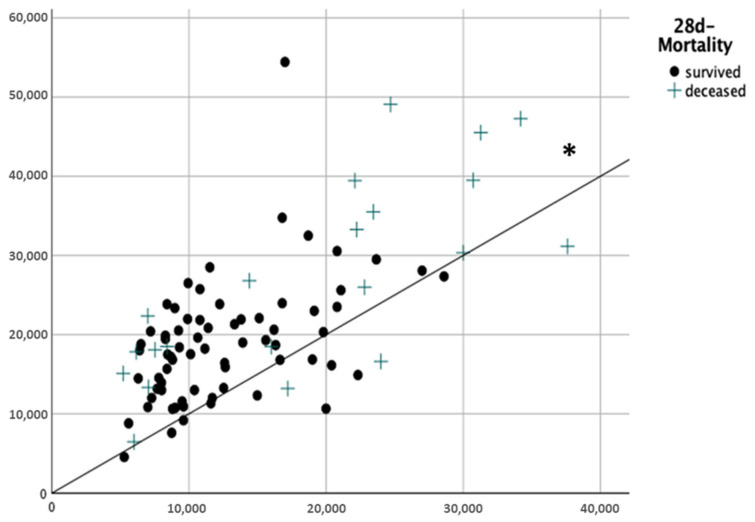
Total amount of fluids applied during burn shock 24 h after trauma. *x*-axis: calculated fluid amount according the Parkland–Baxter formula (mL) *y*-axis: real administered fluids (mL) * *p* < 0.001.

We included 90 severely burnt patients (male/female 60/30, median age 52 (37–63) years, median TBSA 36 (25–51)%, median body mass index (BMI) 27 (23–31) kg/m^2^) (Table 2). The mean amount of fluids applicated in the first 24 h after trauma was 6.5 ±2.5 mL/kgBW%TBSA, which is significantly more than recommended in the Parkland–Baxter formula (Figure 2, *p* < 0.001). A total of 14% (*n* = 13) of the patients received a restrictive fluid therapy (<4 mL/kgBW/%TBSA), 34% (*n* = 31) received a liberal therapy (4–6 mL/kgKG/%TBSA) and 51% (*n* = 46) received an excessive fluid therapy (>6 mL/kgBW/%TBSA).

We found no significant difference concerning gender, age, presence of inhalation- or electrical injury or burn wound severity between groups. Patients in the group with excessive fluid therapy had a significantly lower ABSI score (9 vs. 11, *p* 0.05) and TBSA (35 vs. 51%, *p* < 0.001) than those in the group with liberal fluid therapy. BMI was significantly lower in the group with excessive fluid therapy compared to the liberal (25 vs. 29 kg/m^2^, *p* = 0.01) and restrictive groups (25 vs. 31, *p* < 0.001). The number of surgeries needed to completely close burn wounds was significantly lower with restrictive fluid administration (3.5 vs. 9.0 vs. 7.0, *p* = 0.008). Patients with a fluid resuscitation > 250 mL/kg (positive Ivy index) showed a significantly higher mortality rate (survivors vs. non-survivors: 36 vs. 71%, *p* 0.01) and were more frequently found in the groups with liberal and excessive fluid management (8 vs. 45 vs. 54%, *p* 0.011, Table 3). In the multivariate analysis, the association of the Ivy index and mortality was not significant after adjustment for TBSA and age (OR 2.17, 95% CI 0.55–8.83. *p* = 0.266). A strong correlation between the Ivy index and TBSA (r = 0.62) was observed. Considering pre-existing medical conditions, there was an equal distribution in all groups for coronary heart disease (7.7 vs. 6.5 vs. 6.5%, *p* = 0.987), arterial hypertension (15.4 vs. 29.0 vs. 21.7%, *p* = 0.581), diabetes mellitus (15.4 vs. 12.9 vs. 8.7%, *p* = 0.736) or pulmonary diseases (0 vs. 0 vs. 6.5%, *p* = 0.227). Patients with a history of liver disease (0 vs. 3.2 vs. 13%, *p* = 0.15) or preliminary alcohol abuse (0 vs. 9.7 vs. 21%, *p* = 0.09) tended to be found in the group with excessive fluid administration, but this difference did not reach statistical significance. Patients with pre-existing heart failure were more likely to receive restrictive fluid therapy (23 vs. 3 vs. 4%, *p* = 0.03, Table 4). Patients who achieved criteria of circulatory insufficiency were significantly more often found in the groups with excessive and liberal fluid management than in the restrictive fluid group (89 vs. 90 vs. 61%, *p* = 0.029) but offered no difference in the three groups regarding mortality (72 h mortality 7.7 vs. 6.5 vs. 2.2%, *p* = 0.556; 28 d mortality 23.1 vs. 25.8 vs. 21.7%, *p* 0.918, ICU mortality 38.5 vs. 35.5 vs. 32.6%, *p* = 0.916) or other organ failure (Table 5).

**Figure 3:**
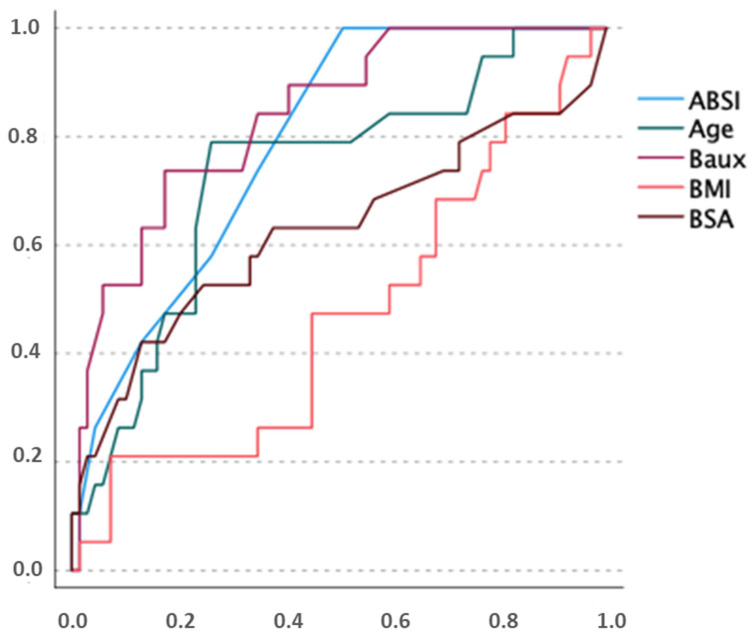
ROC analysis, describing predictive power of our cohort with regard to mortality. *x*-axis: false positive rate *y*-axis: true positive rate.

In our cohort, ABSI, the revised Baux score, age and TBSA significantly predicted death correctly, indicating a study population comparable to other registry studies (Figure 3).

## 4. Discussion

Fluid resuscitation is still the treatment of choice in burn shock, and the focus of research for several years has been on finding the optimum fluid treatment amount and avoiding overtreatment. Despite the well-known recommendations regarding restrictive fluid therapy, in our cohort, more than half of the patients obtained fluids well above the recommended target. One reason for this could be that administration of colloids (human albumin) was only started 24 h after burn shock at the time of this investigation. According to current recommendations, an earlier use of human albumin, i.e., eight hours after injury, is possible. At that time, the capillary leak begins to close. Therefore, this practice might reduce the amount of crystalloid fluids [31,32,33,34].

Saffle et al. reported that one cause of fluid creep is the adjustment of fluid resuscitation not being stringently carried out by the attending physicians. The fluid intake is quickly adapted to the demand by increasing the rate. In contrast, running rates are reduced only very rarely when target parameters are reached [35]. This postulated lack of attention to fluid overload may explain part of the results of our cohort. Patients with minor burn trauma after TBSA and ABSI were often in the group with excessive fluid intake. It can be assumed that patients with less-severe burns were not monitored as closely as patients with high-severity burns with regard to their actual requirements. Interestingly, the literature on fluid overload shows a trend towards overload in patients with a higher burn area [14,36], which we cannot confirm.

There was also no relevant difference in mortality after 72 h and 28 days and overall intensive care mortality between the fluid groups. Organ failures like ARDS, abdominal compartment syndrome or acute kidney injury were not significantly different between the regimes. This lack of significance is noteworthy considering a median fluid administration of almost 8 mL/kgBW/%TBSA. Although the patients with a positive Ivy index > 250 mL/kg were significantly more frequently found in the group with liberal or excess fluid therapy, an association with abdominal compartment syndrome [37] is not reflected in our data. The Ivy index showed a correlation with TBSA in our cohort and is associated with increased mortality [38], so it has to be discussed whether the reason for this is the underlying severity of the burn. The focus must be on finding ways to optimise the fluid amount even with high-percentage burns. Circulatory failure was more frequent in the liberal or excessive fluid groups. This is not unexpected, as it was defined according to norepinephrine dosing (Table 1). One could assume that an increased demand of vasopressors led to a more liberal fluid administration.

Surprisingly, this study does not provide evidence of factors that are usually associated with an excessive resuscitation. For example, neither the proportion of patients with inhalation injury nor of those with full-thickness or high-voltage burns was significantly different in the groups with restrictive, liberal or excessive fluid administration. The share of electrical injury was negligibly low in our cohort, but there was a representative number of patients with deep burns and inhalation injury. For patients with inhalation injury, a fluid requirement of up to 6 mL/kgBW/% TBSA [9] or an additional requirement of 30 mL/kgBW/24 h [39] is described. Controversially, there are studies that do not show an increased need for fluids [36,40]. Therefore, the effect of inhalation injury on increased volume requirements for fluid resuscitation remains unclear and should be the topic of further investigations [40].

Similarly, this seems to be known for patients with full-thickness burns [12,41,42]. We were unable to identify any significant differences in the distribution of patients with full-thickness burns between the groups. There are only very few studies that negate a correlation between burn depth and fluid requirements [43]. One explanation for this could be the generally high amount of fluid applied in our cohort.

There are indications that extensive fluid administration leads to poorer wound healing [44]. Furthermore, the time to complete healing and, thus, the healing of grafts is associated with the amount of initial fluid administration [45]. We can confirm these results. In our cohort, the number of operations until complete coverage was significantly lower in the restrictive group.

There is very little evidence of the influence of pre-existing conditions on the course of fluid resuscitation. The so-called ‘complicated burn resuscitation’ summarises any shock phase complicated by pre-existing conditions or other circumstances such as delayed rescue [46]. As the pathophysiology of long-standing illnesses can influence haemodynamic and fluid responsiveness, this is of considerable relevance for successful resuscitation. In our cohort, we were able to assess the distribution of different pre-existing conditions across the different fluid regimes. We saw trends in the group of patients with a history of alcohol abuse and liver disease towards higher fluid demand, confirming pre-existing data [33,47].

Patients with pre-existing heart failure were found significantly more often in the group with restrictive fluid management. Harrington et al. describe that stress induced by thermal injury filters out patients with insufficient cardiac reserve, resulting in acute-on-chronic heart failure. In addition to the altered haemodynamics with massively increased peripheral vascular resistance and impaired pump function, there is a poorer tolerance of increased preloading [46].

In these special cases, a reduced fluid supply and support of cardiac function via positive inotropic catecholamines could possibly prevent worsening cardiac function. We need to identify those patients who, due to conditions or external causes, have a different fluid requirement than that caused by burn disease.

The results of our investigation show a clear deviation of fluid therapy from the current recommendations. Ultimately, we did not see an increase in serious complications or fatal outcomes. This once again leads to the crucial question of in which patients is an excess or reduced supply of fluid necessary in burn shock.

A fluid administration at the lower limit of requirement quickly leads to obvious symptoms such as increasing vasopressor demands, tachycardia, oliguria and borderline parameters in blood gas analysis. As fluid addition above the targeted limits leads to a short-term easing of a supposedly critical situation but misses the guidelines, we have to train our teams to tolerate borderline results. It is important to distinguish patients with high volume requirements from those for whom an overload is detrimental. Knowledge about pre-existing factors influencing resuscitation can be very helpful.

The limitations of our study are as follows: due to its retrospective nature, there may be an unequal and potentially biased distribution of patients between groups. Furthermore, individual bedside decisions made by the practitioners regarding fluid therapy could not be traced in detail. Whether the high amount of fluids applied in our cohort was the result of a significant increase in demand or a misjudgment cannot be conclusively clarified by this investigation alone.

## 5. Conclusions

Predicting fluid requirements remains difficult. Individualised, timely therapy monitoring over the entire period is as essential as identifying patients with a higher or lower requirement due to various conditions. Fluid overload had impressively fewer deleterious consequences regarding organ failure than expected from known studies but influenced wound healing. Previously published risk factors for higher fluid administration, such as inhalation injury, could not be identified in our cohort. Whereas there are conditions that indicate higher fluid administration, there are also pre-existing factors that should prompt us towards a more restrictive regime. Awareness of the circumstances that lead to a deviation from the usual consumption is helpful. In future, the addition of parameters that provide a clear insight of microcirculation at the bedside might avoid fatal consequences of over- and under-resuscitation.

## Figures and Tables

**Table 1:** Clinical parameters and scores used for definition of organ failure.

Organ Dysfunction/Failure	Score
kidney	Acute kidney injury score (AKI) (serum creatinine/urinary output) [23]
shock	Norepinephrine > 0.1 πg/kg/min for MAP > 65 mmHg [24,25]
liver	King’s College Criteria (INR/serum bilirubin/age/etiology) [26]
	ACLF score [27]
coagulopathy	INR > 1.5 and thrombocytes < 150 Gpt/L [24,28]
lung	Berlin criteria for acute respiratory distress syndrome (PaO_2_/FiO_2_, *x*-ray) [29]
abdominal	
compartment	IAP > 20 mmHg for 24 h and organ dysfunction [30]

**Table 2:** Demographic data.

Number (%) or Median (IQR 25–75) (*n* = 90)		
Sex (%)	female	30 (33.3)
	male	60 (66.7)
Age (years)		52 (37–63)
TBSA (%KOF)		36 (25–51)
Inhalation injury		46 (51.1)
Full-thickness burn		76 (84.4)
Electrocution		2 (2.2)
BMI		27 (23–31)
Baux		104 (86–120)
ABSI		9 (8–11)

**Table 3:** Patient-related factors and association with fluid therapy.

Number (%) or Median (IQR 25–75)						
		All	<4 mL/kgKG/%	4–6 mL/kgKG/%	>6 mL/kgKG/%	*p*
		(*n* = 90)	(*n* = 13)	(*n* = 31)	(*n* = 46)	
Sex (%)	female	30 (33.3)	4 (30.8)	10 (32.3)	16 (34.8)	0.952
	male	60 (66.7)	9 (69.2)	21 (67.7)	30 (65.2)	
Age (years)		52 (37–63)	56 (45–73)	48 (36–62)	54 (39–62)	0.384
TBSA (%KOF)		36 (25–51)	50 (29–60)	50 (34–65)	30 (25–38)	<0.001
Inhalation injury		46 (51.1)	5 (38.5)	16 (51.6)	25 (58.1)	0.453
High-voltage burns		2 (2.2)	1 (7.7)	0 (0)	1 (2.2)	0.287
Full-thickness burns		76 (84.4)	9 (69.3)	26 (83.9)	42 (91.3)	0.128
ABSI		9 (8–11)	10 (9–11)	11 (7–12)	9 (8–10)	0.015
BMI		27 (23–31)	31 (28–33)	29 (26–31)	24 (22–28)	<0.001
Baux		104 (86–120)	106 (94–127)	106 (90–127)	95 (78–116)	0.059
Ivy index (mL/kg)						
<250		50 (55)	12 (92)	17 (55)	21 (46)	0.011
>250		40 (44)	1 (7.7)	14 (45)	25 (54)	
		**all ICU survivors**	**<4 mL/kgKG/%**	**4–6 mL/kgKG/%**	**>6 mL/kgKG/%**	***p***
		(*n* = 59)	(*n* = 8)	(*n* = 20)	(*n* = 31)	
No. of surgeries		7.0 (5.0–11.0)	3.5 (2.0–6.5)	9.0 (6.5–14.5)	7.0 (5.0–9.0)	0.008

**Table 4:** Prevalence of pre-existing diseases among fluid groups.

Number (%)					
	All	<4 mL/kgKG/%	4–6 mL/kgKG/%	>6 mL/kgKG/%	*p*
	(*n* = 90)	(*n* = 13)	(*n* = 31)	(*n* = 46)	
art. hypertension	21 (23.3)	2 (15.4)	9 (29.0)	10 (21.7)	0.581
coronary art. disease	6 (6.7)	1 (7.7)	2 (6.5)	3 (6.5)	0.987
heart failure	6 (6.7)	3 (23.1)	1 (3.2)	2 (4.3)	0.037
diabetes mell.	10 (11.1)	2 (15.4)	4 (12.9)	4 (8.7)	0.736
pulm. disease	3 (3.3)	0 (0)	0 (0)	3 (6.5)	0.227
alcohol abuse	13 (14.4)	0 (0)	3 (9.7)	10 (21.7)	0.093
drugs other	3 (3.3)	0 (0)	2 (6.5)	1 (2.2)	0.455
liver disease	7 (7.8)	0 (0)	1 (3.2)	6 (13.0)	0.152
psych. disease	21 (23.3)	3 (23.1)	7 (22.6)	11 (23.9)	0.991

**Table 5:** Occurrence of organ failures and death in the different fluid groups.

Number (%) or Median (IQR 25–75)					
	All	<4 mL/kgBW/%	4–6 mL/kgBW/%	>6 mL/kgBW/%	*p*
	(*n* = 90)	(*n* = 13)	(*n* = 31)	(*n* = 46)	
Mortality 72 h	4 (4.4)	1 (7.7)	2 (6.5)	1 (2.2)	0.556
Mortality 28 d	21 (23.3)	3 (23.1)	8 (25.8)	10 (21.7)	0.918
Mortality ICU	31 (34.4)	5 (38.5)	11 (35.5)	15 (32.6)	0.916
ARDS	43 (47.8)	5 (38.5)	16 (51.6)	22 (47.8)	0.721
ALF	6 (6.7)	0 (0)	1 (3.2)	5 (11.4)	0.222
Coagulopathy	47 (52.2)	6 (46.2)	15 (48.4)	26 (56.5)	0.699
AKIN	47 (52.2)	7 (53.8)	15 (48.4)	25 (54.3)	0.869
Shock	77 (85.6)	8 (61.5)	21 (90.3)	41 (89.1)	0.029
ACS	14 (15.6)	0 (0)	6 (19.4)	8 (17.4)	0.240

## Data Availability

The original contributions presented in this study are included in the article. Further inquiries can be directed to the corresponding author.

## Abbreviations

3rd-degree burn	full-thickness burn including epidermis and dermis
4th-degree burn	including all skin layers as well as muscles, tendons and bones
ABSI score	abbreviated burn severity index, prognostic score, predicts burn-caused mortality
ACLF	acute-on-chronic liver failure
Baux index	prognostic score, predicts burn-caused mortality
BE	base excess
BMI	body mass index
bpm	beats per minute
BSA	body surface area
FiO_2_	fraction of inspired oxygen
Gpt/L	giga-particles per litre
HR	heart rate
HUO	hourly urinary output
IAP	intra-abdominal pressure
INR	international normalised ratio
MAP	mean arterial pressure
mmHg	millimetres of mercury
PaO_2_	partial pressure of oxygen in arterial blood
PEEP	positive end-expiratory pressure
pH	potential of hydrogen
Pmax	maximum inspiratory airway pressure
POC	point of care testing
Revised Baux score	prognostic score, predicts burn-caused mortality
TTE	transthoracic echocardiography

## References

[1] Daniels M., Fuchs P.C., Lefering R., Grigutsch D., Seyhan H., Limper U., Registry T.G.B., Schiefer J.L. (2021). Is the Parkland formula still the best method for determining the fluid resuscitation volume in adults for the first 24 hours after injury?—A retrospective analysis of burn patients in Germany. Burns.

[2] Paratz J.D., Stockton K., Paratz E.D., Blot S., Muller M., Lipman J., Boots R.J. (2014). Burn resuscitation--hourly urine output versus alternative endpoints: A systematic review. Shock.

[3] Sánchez M., García-de-Lorenzo A., Herrero E., Lopez T., Galvan B., Asensio M.J., Cachafeiro L., Casado C. (2013). A protocol for resuscitation of severe burn patients guided by transpulmonary thermodilution and lactate levels: A 3-year prospective cohort study. Crit. Care.

[4] Pantalone D., Bergamini C., Martellucci J., Alemanno G., Bruscino A., Maltinti G., Sheiterle M., Viligiardi R., Panconesi R., Guagni T. (2021). The Role of DAMPS in Burns and Hemorrhagic Shock Immune Response: Pathophysiology and Clinical Issues. Review. Int. J. Mol. Sci..

[5] Dobson G.P., Morris J.L., Letson H.L. (2024). Pathophysiology of severe burn injuries: New therapeutic opportunities from a systems perspective. J. Burn Care Res..

[6] Rae L., Fidler P., Gibran N. (2016). The Physiologic Basis of Burn Shock and the Need for Aggressive Fluid Resuscitation. Crit. Care Clin..

[7] Baxter C.R., Shires T. (1968). Physiological response to crystalloid resuscitation of severe burns. Ann. N. Y. Acad. Sci..

[8] Holm C., Mayr M., Tegeler J., Hörbrand F., Henckel von Donnersmarck G., Mühlbauer W., Pfeiffer U.J. (2004). A clinical randomized study on the effects of invasive monitoring on burn shock resuscitation. Burns.

[9] Navar P.D., Saffle J.R., Warden G.D. (1985). Effect of inhalation injury on fluid resuscitation requirements after thermal injury. Am. J. Surg..

[10] Dai N.T., Chen T.M., Cheng T.Y., Chen S.L., Chen S.G., Chou G.H., Chou T.D., Wang H.J. (1998). The comparison of early fluid therapy in extensive flame burns between inhalation and noninhalation injuries. Burns.

[11] Culnan D.M., Farner K., Bitz G.H., Capek K.D., Tu Y., Jimenez C., Lineaweaver W.C. (2018). Volume Resuscitation in Patients with High-Voltage Electrical Injuries. Ann. Plast. Surg..

[12] Chen Z., Yuan K. (2011). Another important factor affecting fluid requirement after severe burn: A retrospective study of 166 burn patients in Shanghai. Burns.

[13] Markell K.W., Renz E.M., White C.E., Albrecht M.E., Blackbourne L.H., Park M.S., Barillo D.A., Chung K.K., Kozar R.A., Minei J.P. (2009). Abdominal complications after severe burns. J. Am. Coll. Surg..

[14] Klein M.B., Hayden D., Elson C., Nathens A.B., Gamelli R.L., Gibran N.S., Herndon D.N., Arnoldo B., Silver G., Schoenfeld D. (2007). The association between fluid administration and outcome following major burn: A multicenter study. Ann. Surg..

[15] Mason S.A., Nathens A.B., Jeschke M.G. (2017). “Hold the Pendulum: Rates of Acute Kidney Injury Are Increased in Patients Who Receive Resuscitation Volumes Less Than Predicted by the Parkland Equation”. Ann. Surg..

[16] Arlati S., Storti E., Pradella V., Bucci L., Vitolo A., Pulici M. (2007). Decreased fluid volume to reduce organ damage: A new approach to burn shock resuscitation? A preliminary study. Resuscitation.

[17] Saffle J.I. (2007). The phenomenon of “fluid creep” in acute burn resuscitation. J. Burn Care Res..

[18] Dittrich M.H.M., Hosni N.D., de Carvalho W.B. (2020). Association between fluid creep and infection in burned children: A cohort study. Burns.

[19] Engrav L.H., Colescott P.L., Kemalyan N., Heimbach D.M., Gibran N.S., Solem L.D., Dimick A.R., Gamelli R.L., Lentz C.W. (2000). A biopsy of the use of the Baxter formula to resuscitate burns or do we do it like Charlie did it?. J. Burn Care Rehabil..

[20] Cartotto R., Zhou A. (2010). Fluid creep: The pendulum hasn’t swung back yet!. J. Burn Care Res..

[21] Burmeister D.M., Smith S.L., Muthumalaiappan K., Hill D.M., Moffatt L.T., Carlson D.L., Kubasiak J.C., Chung K.K., Wade C.E., Cancio L.C. (2021). An Assessment of Research Priorities to Dampen the Pendulum Swing of Burn Resuscitation. J. Burn Care Res..

[22] Osler T., Glance L.G., Hosmer D.W. (2010). Simplified estimates of the probability of death after burn injuries: Extending and updating the baux score. J. Trauma Acute Care Surg..

[23] Khwaja A. (2012). KDIGO clinical practice guidelines for acute kidney injury. Nephron Clin. Pract..

[24] Lambden S., Laterre P.F., Levy M.M., Francois B. (2019). The SOFA score-development, utility and challenges of accurate assessment in clinical trials. Crit. Care.

[25] Shankar-Hari M., Phillips G.S., Levy M.L., Seymour C.W., Liu V.X., Deutschman C.S., Angus D.C., Rubenfeld G.D., Singer M. (2016). Developing a New Definition and Assessing New Clinical Criteria for Septic Shock: For the Third International Consensus Definitions for Sepsis and Septic Shock (Sepsis-3). JAMA.

[26] O’Grady J.G., Alexander G.J., Hayllar K.M., Williams R. (1989). Early indicators of prognosis in fulminant hepatic failure. Gastroenterology.

[27] Sarin S.K., Kedarisetty C.K., Abbas Z., Amarapurkar D., Bihari C., Chan A.C., Chawla Y.K., Dokmeci A.K., Garg H., Ghazinyan H. (2014). Acute-on-chronic liver failure: Consensus recommendations of the Asian Pacific Association for the Study of the Liver (APASL) 2014. Hepatol. Int..

[28] Ball R.L., Keyloun J.W., Brummel-Ziedins K., Orfeo T., Palmieri T.L., Johnson L.S., Moffatt L.T., Pusateri A.E., Shupp J.W. (2020). Burn-Induced Coagulopathies: A Comprehensive Review. Shock.

[29] Ranieri V.M., Rubenfeld G.D., Thompson B.T., Ferguson N.D., Caldwell E., Fan E., Camporota L., Slutsky A.S. (2012). Acute respiratory distress syndrome: The Berlin Definition. JAMA.

[30] Malbrain M.L., Cheatham M.L., Kirkpatrick A., Sugrue M., Parr M., De Waele J., Balogh Z., Leppäniemi A., Olvera C., Ivatury R. (2006). Results from the International Conference of Experts on Intra-abdominal Hypertension and Abdominal Compartment Syndrome. I. Definitions. Intensive Care Med..

[31] Cartotto R., Burmeister D.M., Kubasiak J.C. (2022). Burn Shock and Resuscitation: Review and State of the Science. J. Burn Care Res..

[32] Cartotto R., Callum J. (2012). A review of the use of human albumin in burn patients. J. Burn Care Res..

[33] Yowler C.J., Fratianne R.B. (2000). Current status of burn resuscitation. Clin. Plast. Surg..

[34] Navickis R.J., Greenhalgh D.G., Wilkes M.M. (2016). Albumin in Burn Shock Resuscitation: A Meta-Analysis of Controlled Clinical Studies. J. Burn Care Res..

[35] Saffle J.R. (2016). Fluid Creep and Over-resuscitation. Crit. Care Clin..

[36] Cancio L.C., Chávez S., Alvarado-Ortega M., Barillo D.J., Walker S.C., McManus A.T., Goodwin C.W. (2004). Predicting increased fluid requirements during the resuscitation of thermally injured patients. J. Trauma Acute Care Surg..

[37] Ivy M.E., Atweh N.A., Palmer J., Possenti P.P., Pineau M., D’Aiuto M. (2000). Intra-abdominal hypertension and abdominal compartment syndrome in burn patients. J. Trauma Acute Care Surg..

[38] Chung K.K., Wolf S.E., Cancio L.C., Alvarado R., Jones J.A., McCorcle J., King B.T., Barillo D.J., Renz E.M., Blackbourne L.H. (2009). Resuscitation of severely burned military casualties: Fluid begets more fluid. J. Trauma Acute Care Surg..

[39] Inoue T., Okabayashi K., Ohtani M., Yamanoue T., Wada S., Iida K. (2002). Effect of smoke inhalation injury on fluid requirement in burn resuscitation. Hiroshima J. Med. Sci..

[40] Xiao S., Pan Z., Li H., Zhang Y., Li T., Zhang H., Ning J. (2024). The impact of inhalation injury on fluid resuscitation in major burn patients: A 10-year multicenter retrospective study. Eur. J. Med. Res..

[41] Prat N.J., Herzig M.C., Kreyer S., Montgomery R.K., Parida B.K., Linden K., Scaravilli V., Belenkiy S.M., Cancio L.C., Batchinsky A.I. (2017). Platelet and coagulation function before and after burn and smoke inhalation injury in sheep. J. Trauma Acute Care Surg..

[42] Cartotto R.C., Innes M., Musgrave M.A., Gomez M., Cooper A.B. (2002). How well does the Parkland formula estimate actual fluid resuscitation volumes?. J. Burn Care Rehabil..

[43] Ning F., Jiang H., Qiu J., Wang L. (2022). Different Depths May Not Determine the Fluid Resuscitation Volume in Early-stage Management of Severe Burns: A Model-comparison Retrospective Analysis of Fluid Volume Determining Factors. J. Burn Care Res..

[44] Cotton B.A., Guy J.S., Morris J.A., Abumrad N.N. (2006). The cellular, metabolic, and systemic consequences of aggressive fluid resuscitation strategies. Shock.

[45] Nitzschke S.L., Aden J.K., Serio-Melvin M.L., Shingleton S.K., Chung K.K., Waters J.A., King B.T., Burns C.J., Lundy J.B., Salinas J. (2014). Wound healing trajectories in burn patients and their impact on mortality. J. Burn Care Res..

[46] Harrington D.T. (2016). Complicated Burn Resuscitation. Crit. Care Clin..

[47] Abla H., Tran V., Pang A., Stroever S., Shaw C., Dissanaike S., Griswold J. (2024). Assessing resuscitation in burn patients with varying degrees of liver disease. Burns.

